# OLDER ADULTS’ EXPERIENCES OF LONELINESS AND ITS UNDERLYING MECHANISMS IN RESIDENTIAL CARE FACILITIES

**DOI:** 10.1093/geroni/igae098.3588

**Published:** 2024-12-31

**Authors:** Amarjot Gill, Suzanne Sullivan

**Affiliations:** University at Buffalo, SUNY, Buffalo, New York, United States; State University of New York Upstate Medical University, Syracuse, New York, United States

## Abstract

Older Adults’ (OAs’) transition into residential care facilities (RCFs) detaches them from their meaningful social relationships, often precipitating feelings of loneliness. This prevalent issue in RCFs can lead to poor health outcomes, significantly disrupting OAs’ well-being and quality of life. Consequently, loneliness in RCFs emerges as a serious public health issue, necessitating a comprehensive understanding of its mechanism. This systematic review aims to explore OAs’ experiences and perceptions of loneliness and characterize mechanisms underlying OAs’ loneliness experiences through a comprehensive loneliness model. This review adhered to PRISMA guidelines. Articles from CINAHL, PubMed, PsycINFO, and Web of Science, published in English between 2018 and 2023, were examined. Of the 7,285 articles identified, 10 met the inclusion criteria. Quality appraisal for the selected studies was conducted using the Critical Appraisal Skills Programme (CASP) checklist. Three core themes of loneliness were identified: relational and individualized loneliness experiences, perception and emotional distress, and the influence of context and cognitive processes in modulating loneliness. A conceptual model delineating the mechanisms of OAs’ loneliness in RCFs was developed. This review highlights the influence of OAs’ residential context and cognitive processes, particularly their perceptions, in triggering loneliness and associated behavioral responses. OAs’ perceptions of reduced social control and insufficient social connections in RCFs exacerbate their loneliness, precipitating distressing emotional responses and diminished quality of life. Future research must explore strategic transformations of OAs’ perceptions through mechanistic targets and tailored care plans to reshape their social expectations within the context of RCF, potentially mitigating loneliness.

